# Evaluating Two Oral Health Video Interventions with Early Head Start Families

**DOI:** 10.1155/2013/437830

**Published:** 2013-10-29

**Authors:** Lynn B. Wilson, Barbara DeBaryshe, Malkeet Singh, Sharon Taba

**Affiliations:** ^1^Webfish Pacific, LLC, 1188 Bishop Street, Suite 1502, Honolulu, HI 96813, USA; ^2^Center on the Family, College of Tropical Agriculture and Human Resources, University of Hawai'i at Mānoa, Honolulu, HI 96822, USA; ^3^Education Northwest, Center for Research, Evaluation, and Assessment, 101 SW Main Street, Suite 500, Portland, OR 97204, USA; ^4^Department of Pediatrics, John A. Burns School of Medicine, University of Hawai'i at Mānoa, 1319 Punahou Street, Seventh Floor, Honolulu, HI 96826, USA

## Abstract

Poor oral health in early childhood can have long-term consequences, and parents often are unaware of the importance of preventive measures for infants and toddlers. Children in rural, low-income families suffer disproportionately from the effects of poor oral health. Participants were 91 parents of infants and toddlers enrolled in Early Head Start (EHS) living in rural Hawai'i, USA. In this quasi-experimental design, EHS home visitors were assigned to use either a didactic or family-centered video with parents they served. Home visitors reviewed short segments of the assigned videos with parents over an eight-week period. Both groups showed significant prepost gains on knowledge and attitudes/behaviors relating to early oral health as well as self-reported changes in family oral health routines at a six-week followup. Controlling for pretest levels, parents in the family-centered video group showed larger changes in attitudes/behaviors at posttest and a higher number of positive changes in family oral health routines at followup. Results suggest that family-centered educational videos are a promising method for providing anticipatory guidance to parents regarding early childhood oral health. Furthermore, establishing partnerships between dental care, early childhood education, and maternal health systems offers a model that broadens potential reach with minimal cost.

## 1. Introduction

The U.S. Surgeon General released the *Report on Oral Health in America* in 2000 celebrating progress in improving overall oral health nationwide; the report also identified a “silent epidemic” of poor oral health that disproportionately affects vulnerable populations [1, 2]. Low-income children living in rural communities are especially vulnerable to systemic limitations in accessing oral health services including: (1) a lack of access to care—for example, financial, geographical; (2) decreasing numbers of dental providers in proportion to families who need services; (3) a lack of continuity of care involving obstetricians, pediatricians, family physicians, and, for children with special health care needs, specialists; and, (4) low levels of parent and family oral health literacy [1, 3–9]. 

Poor oral health in early childhood can compromise the functional capacity of children to eat, sleep, and learn to speak properly [1]. Moreover, it can lead to mouth pain, inappropriate use of over-the-counter medications, reduced concentration in preschool and school, missed days of school and parental work, expenses associated with childcare or unpaid work leave, overreliance on emergency department resources, cost of hospital admission, and morbidity from general anesthesia, and, in extreme cases, cause infection that reaches the brain resulting in early death [1, 8, 10]. Finally, poor oral health in early childhood has been linked to greater risk of poor oral health later in life; adults with poor oral health have increased risk for coronary heart disease [1, 11]. 

Given these realities, preventing poor oral health in early childhood has become a major emphasis of national and state entities concerned with child health and wellbeing [10, 12]. Because infants and toddlers are not able to care for their own oral health, parents and caregivers play a pivotal role in supporting early oral health—by establishing positive oral health care family routines, implementing proper nutrition, ensuring appropriate amounts of fluoride, and taking children to the dentist by age one [13, 14]. The prevalence of early childhood caries suggests that parents and caregivers lack information about the importance of early oral health and preventing poor oral health [15]. Research on national initiatives, including the Office of Head Start Dental Home Initiative, identifies parent/caregiver education on oral health as a key component in making positive impacts on young children's oral health [16–18]. Developing effective preventive educational “upstream” solutions for oral health can ameliorate social and economic costs to families and communities (cf. Seale and Casamassimo [8], Brown [19], Chasnoff [20]).

This leads to the question of how to most effectively provide parent education and anticipatory guidance. Research on parent education demonstrates that video interventions are more effective than printed pamphlets effecting positive changes in short-term attitudes and reported behaviors and in long-term retention [21]. Adult education research suggests that peer-to-peer approaches, building on social learning theories (cf., Bandura [22, 23]), are more effective in achieving increased knowledge and positive changes in attitudes and behaviors than lecture-based, didactic educational strategies [22, 23]. Social learning theories posit that adults learn best from messages delivered by people whom learners can relate to, emphasizing cooperative learning and peer sharing (e.g., as discussed by Clements and Buczkiewicz [24], Sloane and Zimmer [25], Broadhead et al. [26], Ayala et al. [27], and Leonard-Bartone and Rogers [28]).

The *Baby's First Smiles: Pass It On* video provides an alternative to didactic approaches by integrating peer-to-peer strategies with family-centered approaches (cf. family-centered Medical Home [29, 30]). Medical Home family-centered care, originally developed in the U.S. to effectively serve children with special needs and their families, has now been adopted by the American Academy of Pediatrics for all children [31–34]. The medical home and subsequently the dental home models promote respect for parents as first teachers of and experts on their children, positive family-professional partnerships, and effective community collaboration [30, 31, 34–36]. This enhanced relationship between parents and providers is a key aspect of quality care for young children and their families [35, 36].

Early Head Start (EHS) provides programs to low-income families with young children totaling approximately 150,000 pregnant women and children age birth to three nationwide [37, 38]. For this research in Hawai'i, we engaged EHS home visitors, often seen by families as friends and trusted allies, to share oral health videos with family participants and other family members making the video intervention a more personal, interactive experience. Following the families' video intervention with the home visitor, we asked family participants to share the videos with family members, friends, and community circles employing a peer-to-peer “pass it on” strategy based on trusted personal relationships.

The Pacific island state of Hawai'i has some of the worst child oral health outcomes in the United States partly due to a lack of fluoridation in community water, lack of a state-sponsored dental sealant program, legal limitations preventing dental hygienists from administering fluoride varnish without the direct oversight of a dentist, and shortage of dental providers who accept Medicaid patients [39, 40]. Hawai'i is one of four states in the U.S. with the poorest Medicaid reimbursement to physicians for early dental care (dental exam, anticipatory guidance, and fluoride varnish application) to children under three years of age [40]. Hawai'i is one of five states that received an “F” in an ongoing national assessment of dental health and access to care for disadvantaged children [40].

This evaluation compared family responses to two different approaches to oral health video interventions implemented by Early Head Start Home Visitors for low-income families with young children living in rural Hawai'i: (1) a state-of-the-art didactic video, *Baby's Oral Health*: *Pregnancy Through Childhood* (BOH) produced by the School of Dentistry, University of Toronto [41, 42]; and, (2) *Baby's First Smiles*: *Pass It On* (BFS) produced by Webfish Pacific, LLC [43]. This research hypothesized that family-centered, peer-to-peer videos would be more effective than didactic, lecture-based videos in achieving positive changes in family knowledge, attitudes and behaviors related to young children's oral health among families with young children living in rural Hawai'i.

## 2. Materials and Methods

### 2.1. Oral Health Education Videos

This research project compared two video interventions related to oral health education for parents and caregivers of young children.  Table 1 provides a descriptive comparison of the two videos used in the evaluation, and Table 2 lists the titles of topics in each of the videos.

### 2.2. Participants

Participants were 104 parents of children enrolled in one of two EHS programs serving rural areas of the islands of O'ahu and Hawai'i (the state of Hawai'i is an island chain that includes seven major populated islands; Hawai'i island (aka the Big Island) has the largest landmass while O'ahu has the largest population). The recruitment pool included all families who had been active in EHS for at least four months and were not expecting to move out of their program's service area during the study period. Ninety-one parents (87%) completed the intervention and posttest interviews. Seventy-six parents with posttest data (86%) also participated in a followup interview. Roughly two-thirds of participants lived on O'ahu (68%) and one-third lived on Hawai'i. Among those participants that completed both pre- and posttest interviews, 53% saw the BFS video and 47% saw the BOH video. Almost all participants (96%) were mothers; the sample also included two grandmothers and two fathers. Forty-five percent of families were of Native Hawaiian/Pacific Islander heritage, 27% were of mixed ethnicity, 14% were Asian American, 6% were Caucasian, and less than 4% each were Latino, Native American, or African American. Slightly more participants were married (46%) than single (45%) and 8% were separated or divorced. Twenty-four percent of households spoke a foreign language at home. The modal level of education was a high school diploma or GED (38%) while 15% had less than a high school education, 33% had some college experience, and 13% had a college degree.

Participants had an average of 2.45 children overall, and 1.30 children in the age range served by EHS. Most families (74%) had one child enrolled in EHS, 23% had two children in EHS, and 3% had three enrolled children. Fifty-two percent of the EHS children were boys and 48% were girls. In terms of age distribution, 11% were younger than six months, 16% were 6–12 months, 43% were 13–24 months, and 29% were 25–36 months.

### 2.3. Procedures

Initial discussions were held with all three EHS programs in the state of Hawai'i; one program was not able to participate during the intended time period of the study. Eligible staff participants were home visitors with six or more months experience in their position and who had a caseload of six or more families living in rural communities. Nineteen home visitors (100% of those eligible) agreed to participate. A quasi-experimental design was used where home visitors from both EHS programs (and all the families they served) were randomly assigned to use either the BOH or BFS videos. 

 Home visitors received a three-hour training session. Content included background information on risk factors for poor early oral health, viewing the assigned video, practice showing video topics on portable DVD players, and instructions for following the research protocol. Home visitors were asked to use the assigned video with all consented families they served. For the purpose of intervention design, each video was divided into eight segments lasting four to seven minutes in duration. The segments were to be shown on an overlapping schedule: segment 1 on week one, segments 1 and 2 on week two, segments 2 and 3 on week three, and so forth. Home visitors were asked to complete the series within eight to ten weeks. During the video intervention period, home visitors continued to implement the ongoing EHS protocol. How and when to incorporate the video during each home visit was left to the home visitor's discretion. To minimize differences in presentation, home visitors were asked to simply play the video and to provide only brief answers to any questions from family members. Home visitors were asked to refrain from showing the nonassigned video or from designing and implementing any supplementary curriculum. When each family had completed the video series, the home visitor was asked to give the participant a copy of the assigned video and encourage the family to share the video with other parents in their social circle.

 At the time EHS home visitors were trained, all families were served via a home visiting model. However, both EHS programs had preexisting plans to offer a group-based option to enrolled families. Overall 11 families (12%) decided to move to the group format. To maintain equivalence across home and group formats, staff were asked to show the videos one-on-one to families who opted for group services; this was done either before or after regularly scheduled program activities.

Home visitors were provided with DVD players, assigned DVD videos, and recruitment and record-keeping materials. Home visitors kept a viewing log for each participating family. Logs were submitted to the research team on a monthly basis; the team was also available for consultation via email at any time. Pre- and posttest structured interviews were conducted in person with each participant by a research team member who was blind to the participants' condition. Research team members obtained informed consents from family participants on first contact at the time of the pretest. Posttest interviews were conducted within two weeks of completing the video series. A telephone followup interview was conducted six to eight weeks later. Compensation of $25 per interview was provided to participants; home visitors received a gift of an educational video on early childhood development, and the EHS programs received a combination of cash and material donations worth about $1,000.

### 2.4. Measures

Interview items developed for this study were partly adapted from pediatric and dental research and reviewed by the project's dental advisors [12, 15, 16, 41, 44, 45]. Pre- and posttest interviews included four sections: family demographic characteristics (10 items), oral health knowledge (21 items—see Table 3), and oral health attitudes and behaviors (25 items—see Table 4). The posttest interviews also included a consumer satisfaction scale (8 items—see Table 5). At followup, parents were asked whether their family had made any changes in nutrition habits, oral health routines, or dentist visits, and whether they had shared the video with others (4 items—see Table 6).

The 21 oral health *knowledge* items were presented in a multiple-choice format. Items were written to address the content areas of dental development and professional care (e.g., the role of baby teeth, use of fluoride, and recommended age at first dental visit), family routines in oral hygiene (e.g., wiping gums), and nutrition related to oral health (e.g., effects of different drinks and snacks on preventing/causing tooth decay). The total number of items answered correctly was used to measure overall knowledge. Internal consistency was adequate; coefficient alpha was 0.73 at pretest and 0.78 at posttest. See sample items in Table 3.

The 25 oral health *attitudes/behavior* items were answered using a Likert scale format (1 = *strongly disagree* to 5 = *strongly agree*). Again, content was designed to address dental development, family routines in hygiene, and nutrition. However, the focus was on attitudes and concrete behaviors. Items were reverse coded when needed so that a high score indicated responses supportive of good oral health. Items were summed to form an overall score. Coefficient alphas were 0.82 and 0.88 at pre- and posttest, respectively. See items in Table 4.

The six satisfaction items were answered on a five-point Likert scale (1 = *strongly disagree* to 5 = *strongly agree*). Coefficient alpha was 0.94.

## 3. Results and Discussion

### 3.1. Results

Descriptive statistics for pretest, posttest, and followup data are shown in Table 7. At pretest, parents had the least knowledge about the significance of white spots on the teeth, the development and role of primary teeth, and the recommended age to start flossing and dental checkups (items ranged from 25% to 58% correct) [46–48]. (While there may be a lack of scientific evidence in dental research indicating that flossing teeth prevents cavities, the American Dental Association advocates that flossing “is an essential part of any oral health care routine” and “recommends flossing at least once a day to achieve optimal oral health” [46], and scientific research in early childhood and brain development has firmly established that learning positive behaviors early has long-term benefits [47–49].) Parents were most knowledgeable at pretest about the role of sugar and starches in promoting caries, the need to brush children's teeth, and the need to wipe a baby's gums (items ranged from 84% to 93% correct). Matched pairs *t*-tests were used to test for the main effects of change over time. Knowledge scores increased significantly from pretest (mean = 15.19 or 72% correct) to posttest (mean = 16.98 or 81% correct), *t*_(91)_ = 1.79, *P* < 0.0005, Cohen's *d* = 0.52. Significant gains were also found for the attitude/behavior scale, (means = 103.70 and 107.02 at pre- and posttest, resp.), *t*_(90)_ = 3.31, *P* < 0.0005, and *d* = 0.31. The relative effect sizes (about one-third and one-half standard deviations, resp.) suggest that changes in knowledge were greater than changes in attitudes/behavior.

Group comparisons were conducted to test the hypothesis that the family-focused BFS video would be more appealing to parents and more effective. To adjust for each parent's level of pretest knowledge and attitudes, data were analyzed using a one-way analysis of covariance, with video as the between subjects factor and pretest score as a covariate. (The initial analytic plan was to use multilevel modeling, to account for the nesting of parents under EHS staff. However, preliminary analysis revealed that the intraclass correlation coefficients were close to zero, indicating that there was no need to model shared variance due to EHS staff.) There were no significant group differences on knowledge or attitudes/behavior at pretest, although there was a trend in the direction of BFS parents having higher pretest knowledge scores. Controlling for pretest knowledge, there were no posttest differences in knowledge scores, *F*_(1,88)_ = 0.01, *P* < 0.91, and *d* = 0.02. The BFS group had higher scores for oral health attitudes/behaviors at posttest, *F*_(1,88)_ = 3.96, *P* < 0.05; this effect was modest in magnitude, *d* = 0.27.

A two-group analysis of variance was used to test for group differences at posttest on consumer satisfaction and for group differences at followup on changes in oral health behavior. The BFS group had higher satisfaction scores (means = 34.35 and 36.83 for BOH and BFS, resp.), *F*_(1,89)_ = 7.50, *P* < 0.007, and *d* = 0.57. BFS families also reported a higher number of changes in family oral health routines, nutritional practices, and dentists visits (means = 2.09 and 3.27 changes, resp.), *F*_(1,76)_ = 5.82, *P* < 0.02, and *d* = 0.55. 

A content analysis was conducted on parents' open-ended responses. When asked to list the types of changes their family had made since watching the videos, the most common changes mentioned were (a) increased frequency, duration, or attention to brushing children's teeth, (b) decreasing intake of sugary foods, snacks, and drinks, (c) increasing consumption of fruits and vegetables, (d) increasing the frequency of flossing children's teeth, and (e) increases in actual visits or the intention of taking the child to the dentist. Some changes that were mentioned only by the BFS group included parents or siblings serving as role models of oral hygiene, increasing water consumption, increases in the parents seeing the dentist, attention to fluoride, and changes in bottle feeding. These group differences are consistent with the content of each video series, that is, the BFS series suggests that children should watch their parents brush and floss and mentions repeatedly the importance of fluoride. 

### 3.2. Discussion

The early childhood period is one of relatively untapped potential for setting positive lifelong oral health practices and preventing later oral health problems. However, access to parents for the purposes of anticipatory guidance is often limited, particularly parents of infants and toddlers. Parents of very young children may be unaware of the need for early oral hygiene and dental examination schedules. If a family lacks dental insurance, there may be few opportunities for a child to receive dental screening unless such services are provided in their childcare, preschool, or elementary school settings.

Parent education via short, realistic videos is a low-cost and potentially engaging avenue for disseminating information. Visual media also have the advantage of being effective regardless of levels of literacy among the target population. Results of this study suggest that educational videos can be effective, at least in the short term. Parents of children age birth to three showed small but statistically significant increases in knowledge, attitudes, and self-reported family oral health practices after seeing segments of the BOH or BFS video series over an eight-week period.

Two questions regarding the delivery of media-based anticipatory guidance are (a) does the presentation style of the video itself make a difference, and (b) how can more families be exposed to video-based education? In terms of presentation style, the results of this study indicate that a family-centered approach (BFS) is more effective than the more traditional didactic approach (BOH). A didactic approach focuses on facts and suggested practices, with narration provided by professional voice-over and/or by interviews with authority figures. While visuals may still be realistic and engaging, the tone tends to be more formal and the audience is positioned as the recipient of the experts' advice. A family-centered approach is still research based, but the tone is more informal, and peers act as messengers who share their personal experiences and strategies for implementing what the experts suggest should be done. In this experimental study, we found that didactic (BOH) and family-focused (BFS) styles resulted in similar gains in factual knowledge. However, the family-focused video (BFS) resulted in higher consumer satisfactions and greater parental attitudinal and self-reported behavioral change. This suggests that the family-focused style is more effective.

Delivery strategies for anticipatory guidance are a second key consideration. While short videos such as the ones used in this study are inexpensive and feasible for use in pediatric dental practices and medical clinics (where parents can watch video segments in the waiting area), the model followed in this study was to capitalize on the use of an existing system of early childhood service providers. In this study, we asked Early Head Start (EHS) home visitors and group leaders to disseminate the video to the families they serve. This model has the potential to reach many more children, especially those who are not already receiving early pediatric dental care. In the U.S., over 150,000 low-income children enroll in EHS on an annual basis [49]. Over 6.4 million infants and toddlers and their mothers receive nutritional subsidies and education through the Women, Infants, and Children (WIC) program, and roughly one million children three through five years of age receive preschool services through Head Start [16, 50]. The majority of states in the U.S. now offer targeted or even universal public preschool [51]. Programs like EHS, WIC, Head Start, and state-funded preschool provide a fruitful avenue for accessing parents of young, at-risk children. In other countries, organizations with widespread reach may include national childcare and preschool programs, maternal and child health nurses, and home visitors. 

In addition to the number of children served, relying on early childhood educators and maternal and child health care providers as a conduit of information has other potential benefits. These personnel (especially early childhood educators) often have close and long-term personal relationships with the children and families they serve. There is a level of trust and knowledge of child development that allows for open discussion, individualized advice, and followup. There is also the element of time, where teachers and home visitors are not working within a schedule of short appointments and long waiting times. In early childhood settings, attending the program is already part of the family's regular routine, and there is also the expectation that staff work actively to support parents' positive influence on the child's overall development and wellbeing. 

This study has several limitations. First, the BFS and BOH videos differed on characteristics such as overall length and some aspects of informational content as well as presentation style. For this reason, differences cannot be attributed solely to the didactic versus family-focused presentation style. The sample size was modest and was taken from a small geographic area. Most importantly, outcomes were all based on parent report, and no objective measures of changes in family behavior or children's oral health were collected. Finally, the followup period was relatively short. However, the results provide an informative first step in developing new materials and dissemination models for educating parents about early oral health.

## 4. Conclusions

Family-focused educational videos are a promising method for providing anticipatory guidance to parents of infants, toddlers, and preschoolers regarding early childhood oral health. Furthermore, supporting partnerships among dental care and early childhood education and maternal health systems represents a model that offers wide potential reach with minimal cost.

## Figures and Tables

**Table 1:** Features of the *Baby's Oral Health* and *Baby's First Smiles* videos.

	Didactic video Baby's Oral Health (BOH)	Family-centered video Baby's First Smiles (BFS)
Duration	19 min.	26 min.

Age Range	Prenatal through elementary	Prenatal through age three

Messaging	(1) Research-based messages, best practices	(1) Research-based messages, best practices
	(2) Lecture format	(2) 1st person interview format of families with young children and family-centered professionals
	(3) Offscreen narrator for entire video	(3) Interviewee messages and strategies emerged from interviews (parents provided 70% of messages, dentists provided 30% of messages)
	(4) Script written based on didactic points written in a dentist's voice	(4) Emphasis on peer-to-peer learning
		(5) Emphasis on child development and family context
		(6) Emphasis on life-long consequences

Visuals	(1) High quality video capturing visual examples of the messages from offcamera narrator	(1) High quality video capturing interviews with multiple parents and family-centered pediatric dentists including visual examples of the messages covered in interviews that focus on parents, families, and young children
		(2) Interviews with families conducted in their homes and show daily routines
		(3) Animations begin every topic

**Table 2:** Topical organization of video content.

Didactic video Baby's Oral Health (BOH) University of Toronto School of Dentistry	Family-centered video Baby's First Smiles (BFS) Webfish Pacific, LLC Early Childhood Oral Health Initiative
(1) The role of a healthy pregnancy in the development of baby teeth (2) Stages of development of baby teeth (3) Healthy nutrition for healthy baby teeth (4) Oral hygiene (5) Benefits and proper use of fluoride (6) Source of bacteria in the mouth (7) Night feeding habits (8) Early baby tooth decay (9) Oral habits (10) Prevention of injuries (home, play area, and car safety) (11) The baby's first dental visit (12) Regular dental visits	(1) What the tooth fairy forgot to tell us! (2) Starting early (3) Healthy mouths (4) Junk mouths (5) Family matters (6) Healthy eating (7) Going to the dentist (8) Community partners

**Table 3:** Sample pre- and posttest knowledge questions—multiple choice, 21 items total.

(1) Cleaning baby's mouth after feeding should begin
(a) At birth
(b) After the baby is a year old
(c) After the first tooth comes in
(d) After the baby starts eating solid foods
(2) White spots on a child's teeth means
(a) The child is drinking too much milk
(b) The teeth are not getting clean enough
(c) The teeth will be protected from cavities
(d) The child is eating too much cheese
(3) Giving a baby fruit juice in a bottle at night
(a) Fights bacteria in the mouth
(b) Increases the chances for cavities
(c) Prevents cavities
(d) Prevents white spots from forming on the teeth
(4) Permanent teeth
(a) Are more important than baby teeth
(b) Do not form beneath the gums until the baby teeth fall out
(c) Are not affected by the baby teeth
(d) Form beneath the baby teeth before a child is born
(5) The amount of fluoride a child needs
(a) Depends on the child's age
(b) Depends on the kind of toothpaste they use
(c) Is the same for all children
(d) Is the same as adults
(6) Plaque is
(a) A film of bacteria on teeth
(b) A film that protects teeth from cavities
(c) A film that prevents teeth from yellowing
(d) Holes in the teeth that cause cavities

**Table 4:** Pre- and posttest attitudes/behavior items—5 point Likert Scale, 25 items.

(1) Parents/caregivers need to clean (wipe or brush) their baby's mouth at least twice a day.	
(2) Children should watch parents/caregivers brush their teeth at least once a week.	
(3) I have asked my dentist about the amount of fluoride young children need.	
(4) Parents/caregivers need to provide a variety of food to their children so their children are more likely to try new foods.	
(5) I would feel comfortable wiping a baby's gums.	
(6) I feel that it is important for a pregnant mom to go to the dentist to take care of her own teeth.	
(7) It is good when parents/caregivers reach out to trusted friends for information about the oral health of a young child.	
(8) I have talked to a dentist about when to start flossing young children's teeth.	
(9) I believe children should be served fresh fruits every day.	
(10) I believe parents can prevent their children from having cavities.	
(11) I am uncomfortable when I see someone giving soda to a young child.	
(12) When parents/caregivers brush their teeth twice a day, their children are more likely to brush their teeth twice a day.	
(13) New mothers/parents/caregivers can get support by talking to other new mothers/parents/caregivers in making sure their child's teeth and gums stay healthy.	
(14) I feel that taking a child to the dentist when they are very young helps them not be afraid of the dentist.	
(15) I prefer to give juice to a young child all through the day because it is good for them.*	
(16) Parents/caregivers need to give appropriate amounts of fluoride to their children depending on their age.	
(17) I would put a baby to bed with a bottle that has only milk in it.*	
(18) Parents/caregivers need to look in children's mouths for white spots on their teeth at least once a month.	
(19) I believe a child's first dental visit should be when they feel pain in their mouth.*	
(20) I brush my own teeth at least twice a day.	
(21) I believe children need help in tooth brushing until they are seven years old.	
(22) I feel a young child's first visit to the dentist should be after they are two years old.*	
(23) I avoid going to the dentist whenever I can.*	
(24) Parents/caregivers need to take children to the dentist every six months.	
(25) My goal is to give young children fewer sweet drinks and foods.	

*Indicates reverse order for Likert scale scoring.

**Table 5:** Posttest consumer satisfaction—5 point Likert scale, 8 items.

(1) The oral health videos appealed to me.	
(2) The oral health videos emphasized that all members of the family need to be involved in our children's oral health routines.	
(3) I could relate to the people in the videos.	
(4) I learned a lot from the videos about caring for my baby's mouth starting from birth.	
(5) I would recommend these oral health videos for use in Early Head Start programs.	
(6) I would recommend these oral health videos to the following:	
(a) family members	
(b) friends	
(c) neighbors	

**Table 6:** Followup survey items on self-reported behavior—4 items.

(1) Is there anything different about your family's oral health practices and routines now that you have seen the videos?	
(a) Nothing is changed	
(b) Something is changed—What changed? (List up to 4 changes)	
(2) Is there anything different about your family's eating habits now that you have seen the videos?	
(a) Nothing is changed	
(b) Something is changed—What changed? (List up to 4 changes)	
(3) Is there anything different about your family's schedule of going to the dentist now that you have seen the videos?	
(a) Nothing is changed	
(b) Something is changed—What changed? (List up to 4 changes)	
(4) With how many people have you shared the oral health videos?	
(a) No one	
(b) 1-2 people	
(c) 2–4 people	
(d) 5–9 people	
(e) 10 or more people	

**Table 7:** Means and standard deviations for study measures.

Variable	Time	Group					
		BOH		BFS		Total	
		Mean	SD	Mean	SD	Mean	SD
Knowledge	Pre	15.30	3.38	15.08	3.51	15.19	3.43
	Post^a^	17.09	3.19	16.87	3.65	16.98	3.42

Attitudes/behaviors	Pre	102.91	9.93	104.42	10.08	103.70	9.98
	Post^b^	104.81	9.30	109.00	11.92	107.02	10.91

Satisfaction	Post	34.35	4.54	36.83	4.11	35.66	4.47

Number of changes	Followup	2.09	1.93	3.27	2.26	2.77	2.19

^a^Covariate adjusted posttest means = 17.01 and 16.95 for BOH and BFS, respectively.

^b^Covariate adjusted posttest means = 105.46 and 108.42 for BOH and BFS, respectively.
